# Rapid screening for *Chlamydia trachomatis* infection by detecting α-mannosidase activity in urogenital tract specimens

**DOI:** 10.1186/1471-2334-13-36

**Published:** 2013-01-24

**Authors:** Ze-yu Wang, Guang-yu Fu, Shan-mei Wang, Dong-chun Qin, Zhong-quan Wang, Jing Cui

**Affiliations:** 1 Department of Pathogen Biology, School of Basic Medicine, Zhengzhou University, Zhengzhou, 450001, China; 2 Department of Clinical Laboratory, Henan Provincial People’s Hospital, Zhengzhou, 450003, China; 3 Department of Clinical Laboratory, First Affiliated Hospital, Zhengzhou University, Zhengzhou, 450052, China

**Keywords:** *Chlamydia trachomatis*, α-mannosidase, Activity, Gold standard, Marker

## Abstract

**Background:**

*Chlamydia trachomatis* may cause multiple different urogenital tract disorders, but current non-culture assays for rapid screening of *C. trachomatis* typically use immunochromatography-based methods. We established another new rapid non-culture method for detection of *C. trachomatis* based on the measurement of α-mannosidase enzymatic activity in urogenital tract specimens.

**Method:**

To evaluate the performance of this method, α-mannosidase activities of *C. trachomatis* serotype D strain 、 and 29 standard strains related to clinical urogenital pathogens were investigated. Furthermore, 553 urogenital tract specimens were used for clinical assays via cell culture method and ligase chain reaction method (LCR), adopting an expanded gold standard.

**Results:**

Only *C. trachomatis* was positive for α-mannosidase activity among different types of microbes tested in the research. When prostate fluid specimens, which have some interfering activity, were excluded, the sensitivity and specificity of the enzymatic method were 91.8% (78/85) and 98.3% (409/416), respectively. There were no significant differences (*P* > 0.05).

**Conclusions:**

These results showed that α-mannosidase activity could be utilised as a screening marker of *C. trachomatis* infection.

## Background

*C. trachomatis* infection is the most common sexually transmitted disease (STD) in the United States [1]. Mounting evidence has indicated that it not only evokes nongonococcal urethritis (NGU), cervicitis, pelvic inflammatory disease (PID), salpingitis, orchitis, and epididymitis, but also increases risk of invasive cervical cancer [2,3] and gives rise to serious reproductive disorders such as infertility [4,5], miscarriage/premature birth/missed miscarriage [6-9], and neonatal conjunctivitis [10].

A large number of methods have been established for screening and diagnosis of *C. trachomatis* infection [11]. Nevertheless, few of these assays meet the requirements of outpatient diagnosis, especially in terms of sensitivity, specificity, time, and simplicity of operation. Currently, non-culture assays for *C. trachomatis* screening typically adopt immunochromatography-based methods. Technologies based on chromogenic reactions of specific microbial enzymes have been widely applied in bacterial identification systems and chromogenic media [12-15]. However, no chromogenic assay for detecting *C. trachomatis* has been made available to date.

Our previous findings [16] suggested that *C. trachomatis* might have very high α-mannosidase activity. The purpose of the study was to establish a novel screening method for *C. trachomatis* infection without culture that would be rapid and convenient for use in outpatient clinics.

## Methods

### Ethics statement

All patients were treated in accordance with the Helsinki Declaration on the participation of human subjects in medical research. Ethics approval for the study was obtained from the First Affiliated Hospital Ethics Committee of Zhengzhou University (Approved No. 20100802) and Henan Provincial People’s Hospital Ethics Committee (Approved No. 20100901).

### Organisms

Reference strains and cell lines were obtained from the organisations shown in Table 1.

**Table 1:** Reference strains and cell line

**Strains or cell line**	**Accession numbers**	**Organizations**
*Acinetobacter baumannii*	ATCC19606	Harmony Biotechnology Co., Ltd (Shanghai, China)
*Candida albicans*	ATCC10231	Harmony Biotechnology Co., Ltd (Shanghai, China)
*Candida glabrata*	ATCC15126	Harmony Biotechnology Co., Ltd (Shanghai, China)
*Candida guilliermondii*	ATCC6260	Harmony Biotechnology Co., Ltd (Shanghai, China)
*Candida krusei*	ATCC14243	Harmony Biotechnology Co., Ltd (Shanghai, China)
*Cryptococcus neoformans*	CMCC(F)D2q	China Medical Microbiological Culture Collection Center (fungi)(Nanjing, China)
*Candida parapsilosis*	CGMCC2.1846	China General Microbiological Culture Collection Center (Beijing, China)
*Candida tropicalis*	ATCC750	Harmony Biotechnology Co., Ltd (Shanghai, China)
*Chlamydia trachomatis* Serovar D	VR-885	American Type Culture Collection(Manassas, USA)
*Enterococcus faecalis*	ATCC29212	Huankai Microbial Sci & Tech. Co., Ltd (Guangzhou, China)
*Enterococcus faecium*	ATCC700221	Harmony Biotechnology Co., Ltd (Shanghai, China)
*Escherichia coli*	ATCC25922	Henan Provincial Institute of Food and Drug Control (Zhengzhou, China)
*Gardnerella vaginalis*	ATCC14018	Harmony Biotechnology Co., Ltd (Shanghai, China)
*Haemophilus influenzae*	ATCC10211	Harmony Biotechnology Co., Ltd (Shanghai, China)
*Klebsiella pneumoniae*	CMCC46117	Tianhe Microorganism Reagent Co., Ltd (Hangzhou, China)
McCony	CRL-1696	American Type Culture Collection(Manassas, USA)
*Mycoplasma hominis*	ATCC15488	Harmony Biotechnology Co., Ltd (Shanghai, China)
*Neisseria gonorrhoeae*	ATCC19424	Henan Provincial Center for Disease Control and Prevention (Zhengzhou, China)
*Pseudomonas aeruginosa*	ATCC25619	Land Bridge Biotechnology Co., Ltd (Beijing, China)
*Proteus mirabilis*	CMCC(B)49005	Huankai Microbial Sci & Tech. Co., Ltd (Guangzhou, China)
*Salmonella enteritidis*	ATCC13076	Land Bridge Biotechnology Co., Ltd (Beijing, China)
*Staphylococus aureus*	ATCC25923	Henan Provincial Institute of Food and Drug Control (Zhengzhou, China)
*Staphylococcus aureus*	ATCC29213	Capital Institute of Pediatrics (Beijing, China)
*Staphylococcus epidermidis*	ATCC12228	Huankai Microbial Sci & Tech. Co., Ltd (Guangzhou, China)
*Staphylococus saprophyticus*	ATCC49453	Land Bridge Biotechnology Co., Ltd (Beijing, China)
*Stenotrophomonas maltophilia*	ATCC17666	Land Bridge Biotechnology Co., Ltd (Beijing, China)
*Streptococus agalactiae*	ATCC13813	Harmony Biotechnology Co., Ltd (Shanghai, China)
*Streptococus pneumoniae*	ATCC49619	National Center for Clinical Laboratory (Beijing, China)
*Streptococus pyogenes*	ATCC19615	Harmony Biotechnology Co., Ltd (Shanghai, China)
*Trichomonas vaginalis*	ATCC30001	Harmony Biotechnology Co., Ltd (Shanghai, China)
*Ureaplasma urealyticum*	ATCC15531	Harmony Biotechnology Co., Ltd (Shanghai, China)

### Specimens

This study evaluated 553 specimens from clinical patients attending the STD (257, 46.47%) and Gynaecology (296, 53.53%) clinics at the First Affiliated Hospital of Zhengzhou University (Zhengzhou, China) and the Henan Provincial People’s Hospital (Zhengzhou, China), respectively. For the 203 male cases, three urethral discharge specimens (151 outpatients, 74.38%) or three prostate massage liquid specimens (52 outpatients, 25.62%) were collected with sterile rayon swabs (Copan, Brescia, Italy). Meanwhile, for the 350 female cases, three cervical secretion specimens (232 outpatients, 66.29%) or three vaginal secretion specimens (118 outpatients, 33.71%) were collected with sterile rayon swabs using vaginal forceps. Three swabs collected for each specimen, were no significant differences in sampling link and randomly used with three methods. None of the patients received any antibiotics one week before sample collection, when samples were taken before diagnosis.

### Media, culture and inoculation

Liquid media A (LMA) and liquid media B (LMB) were prepared for mycoplasma culture. The components of LMA were shown in Table 2, but LMB consisted of 50 mg/l phenol red besides LMA components. The media were inoculated with both *Ureaplasma urealyticum* and *Mycoplasma hominis* and analyzed by using the colour-changing unit (CCU) method, as previously reported [17]. Once the concentrations of mycoplasma reached 10^6^ CCU/ml in LMB, the cultures of LMA should be immediately stored at 4°C.

**Table 2:** Composition of LMA for Mycoplasma (per liter) *

**Ingredient**	**Concentration**	**Ingredient**	**Concentration**
NaCl	6.4 g	Beef heart extract power	7.2 g
CaCl_2_	112 mg	Yeast extract power	2.72 g
MgCl_2_·6H_2_O	80 mg	Peptone	8.0 g
MgSO_4_·7H_2_O	80 mg	Horse serum	200 ml
KCl	320 mg	Penicillin sodium	1,000,000 units
Na_2_HPO_4_·12H_2_O	122 mg	Ampicillin sodium	375 mg
KH_2_PO_4_	48 mg	Vancomycin	40 mg
Cysteine hydrochloride	0.8 g	Polymyxin B	40,000 units
Arginine hydrochloride	4.0 g	Nystatin	15,000 USP units
Urea	4.0 g		

*Adjusted to pH 6.25.

*Trichomonas vaginalis* was obtained after 24 h incubation at 37°C in Trichomonas medium (Oxoid, Basingstoke, UK) supplemented with 8% horse blood and 1,000 units/ml penicillin sodium and 500 mg/ml streptomycin. The collection was stored at 4°C.

All other micro-organisms except *C. trachomatis* used in the study were inoculated and cultured as described in Table 3. The collections described in Table 3 were resuspended to 0.5 McFarland standards in a sterile solution of 0.9% NaCl and then stored at 4°C.

**Table 3:** The media and culture methods for bacteria and candida

**Strains**	**Media**	**Temp.**	**Time**
**Bacteria**			
*A. baumannii*, *E. faecalis*,	Blood agar base ^a^	37°C	24 h
*E. faecium*, *E. coli*, *K. pneumoniae*,			
*P. mirabilis*, *P. aeruginosa*,			
*S. enteritidis*, *S. aureus*,			
*S. epidermidis*, *S. saprophyticus*,			
*S. maltophilia*, *S. agalactiae*,			
*S. pyogenes*, *S. pneumoniae*			
*G. vaginalis* *	Blood agar base	37°C	48 h
*H. influenzae* *	Thayer Martin media ^b^		24 h
*N. gonorrhoeae* *			48 h
**Candida**			
*C. albicans*, *C. tropicalis*,	Sabouraud dextrose agar ^c^	37°C	24 h
*C. glabrata*, *C. parapsilosis*,			
*C. guilliermondii*, *C. krusei*,			
*C. neoformans*		30°C	48 h

^a,b,c^ Oxoid, Basingstoke, UK; ^a^ supplemented with 5% sheep blood; ^b^ supplemented with 5% horse blood; * cultured in a candle jar.

### Enzymatic method

The enzymatic method was based on the substrate of α-D-mannosidase. The substrate solution contained 1.5 mg/ml 6-chloro-3-indolyl-α-D-mannoside (J&K, Shanghai, China), 100 mM citrate buffer (pH 4.0), and 1% Triton X-100. The sample diluent was 0.9% NaCl. The chromogenic reagent contained 0.08% fast violet B salt (J&K, Shanghai, China). Aliquots (50 μl) of substrate solution and chromogenic reagent were added sequentially into sample solutions (extracted from every swab sample with 500 μl sample diluent) or the aforementioned microbial suspensions as well as the chlamydial suspension mentioned in the section called limit of detection (LOD) of the enzymatic method. The result was considered to be positive (OD_512_ ≧ 0.150) if the colour changed from colourless to red or brown after 15 min of incubation at 37°C; otherwise, the result was considered to be negative (OD_512_ < 0.150). The suspensions containing bacteria or cells was centrifuged at 5,000 rpm for 5 min, and then OD_512_ value of the supernatants were measured.

### LOD of the enzymatic method

McCoy cells infected with *C. trachomatis* serovar D were stored at −80°C, and frozen-thawed twice to obtain chlamydial suspension before use. Serial 10-fold dilutions of the chlamydial suspension were inoculated in six duplicate into wells (100 μl/well) of cycloheximide-treated McCoy cell monolayers that had been incubated with MEM medium (Gibco, Grand Island, NY, USA) in a 96-well flat-bottom microtiter plate (Nunc Inc., Roskilde, Denmark) at 37°C under 5% CO_2_ for 48 h. Inclusion body titers of the chlamydial supernatant were quantified by titrating the number of inclusion-forming units (IFU). The contents of each well were stained with a *C. trachomatis* direct fluorescent antibody kit (Academy of Military Medical Science, Beijing, China) and examined by microscopy for IFU counts. The average IFU of each dilution culture of three replicate wells was taken as the concentration of *C. trachomatis* in the corresponding dilution of the chlamydia suspension. Each dilution culture of the other three replicate wells was examined via the enzymatic method after collection to determine the LOD for *C. trachomatis* of the enzymatic method. 10^-1^ u/ml α-D-mannosidase (EC3.2.1.24; Sigma, USA) solution was 5-fold serially diluted to 10^-4^ u/ml. These enzyme solutions fold-diluted were detected via the enzymatic method to determine the LOD for α-D-mannosidase.

### Reference method

Cell culture and LCR method were used to evaluate the clinical performance of the enzymatic method. The swabs used for culture were dipped directly into incidental transport medium (Copan, Brescia, Italy) and cultured according to the aforementioned method. The cultures were tested using a *C. trachomatis* direct fluorescent antibody kit. LCR was carried out using the LCx *C. trachomatis* assay (Abbott Laboratories, Abbott Park, Israel) according to the manufacturer’s instructions. Although culture method has a good specificity, its sensitivity may be influenced by various factors. Therefore, the research adopted an “expanded gold standard” [18-20] described as follows: any positive by either culture or LCR was classified as a true positive, whatever the result of the enzymatic method.

## Results

In our developed assay, only *C. trachomatis* samples tested was positive for α-D-mannosidase activity; but OD_512_ of both other organisms and cell cultures which were not inoculated with *C. trachomatis* used in the study was all below 0.100, which fell into the range of negative results negative with 0.150 (OD_512_) as the cut-off value, even if the reactions were allowed to proceed for 1 h at 37°C. Of the 553 clinical samples, 132 samples were positive with OD_512_ ranging from 0.161 to 1.955, and 421 samples were negative with OD_512_ ranging from 0.013 to 0.142. *C. trachomatis* detection results of 553 cases with culture, LCR and enzymatic method used an expanded gold standard as the reference standard were showed in Table 4. The enzymatic method was least reliable when prostate specimens were used. The sensitivity of the enzymatic method was 91.5% (95% confidence interval [CI], 85.9% to 97.1%), and the specificity of this method was 90.0% (95% CI, 87.3% to 92.7%) (Table 5). The sensitivity and specificity of the LCR were 94.7% (95% CI, 90.2% to 99.2%) and 100% (95% CI, 100.0% to 100.0%), respectively. However, in those specimens other than prostate fluid samples, the sensitivity and specificity of the enzymatic method were 91.8% (95% CI, 86.0% to 97.6%) and 98.3% (95% CI, 97.1% to 99.5%), in the meantime the sensitivity and specificity of the LCR were 95.3% (95% CI, 90.8% to 99.8%) and 100% (95% CI, 100% to 100%) (Table 6), respectively. There were no significant differences in performance between the enzymatic method and the expanded gold standard (*P* > 0.05).

**Table 4:** ***C. trachomatis *detection results of 553 cases with culture, LCR and enzymatic method used an expanded gold standard as the reference standard**

**Method**	**No. of true positives/positives**	**No. of true negatives/negatives**
Enzymatic method	86/132	413/421
	Urethral discharge 22/24	Urethral discharge 126/127
	Prostate massage liquid 8/47	Prostate massage liquid 4/5
	Cervical secretion 31/33	Cervical secretion 196/199
	Vaginal secretion 25/28	Vaginal secretion 87/90
Cell culture	60/60	459/493
	Urethral discharge 18/18	Urethral discharge 128/133
	Prostate massage liquid 7/7	Prostate massage liquid 43/45
	Cervical secretion 21/21	Cervical secretion 198/211
	Vaginal secretion 14/14	Vaginal secretion 90/104
LCR	85/89	459/464
	Urethral discharge 22/22	Urethral discharge 128/133
	Prostate massage liquid 8/8	Prostate massage liquid 43/45
	Cervical secretion 32/32	Cervical secretion 198/211
	Vaginal secretion 27/27	Vaginal secretion 90/104
Expanded gold standard	94	459
	Urethral discharge 23	Urethral discharge 128
	Prostate massage liquid 9	Prostate massage liquid 43
	Cervical secretion 34	Cervical secretion 198
	Vaginal secretion 28	Vaginal secretion 90

**Table 5:** **Clinical performances of three assays for *C. trachomatis *using specimens included prostate massage liquid**

**Methods**	**% Sensitivity (95% CI)**	**% Specificity (95% CI)**
Enzymatic method^a^	91.5 (85.9, 97.1)	90.0 (87.3, 92.7)
Cell culture^b^	63.8 (54.1, 73.5)	100.0 (100.0, 100.0)
LCR^c^	94.7 (90.2, 99.2)	100.0 (100.0, 100.0)

^a^ Χ^2^ = 8.030, *P* = 0.005, *P* < 0.05; ^b^ Χ^2^ = 8.721, *P* = 0.003, *P* < 0.05; ^c^ Χ^2^ = 0.164, *P* = 0.685, *P* > 0.05.

**Table 6:** **Clinical performances of three assays for *C. trachomatis *using specimens excluded prostate massage liquid**

**Methods**	**% Sensitivity (95% CI)**	**% Specificity (95% CI)**
Enzymatic method^a^	91.8 (86.0, 97.6)	98.3 (97.1, 99.5)
Cell culture^b^	64.6 (54.3, 74.9)	100.0 (100.0, 100.0)
LCR^c^	95.3 (90.8, 99.8)	100.0 (100.0, 100.0)

^a^ Χ^2^ = 0, *P* = 1, *P* > 0.05; ^b^ Χ^2^ = 8.605, *P* = 0.003, *P* < 0.05; ^c^ Χ^2^ = 0.116, *P* = 0.734, *P* > 0.05.

The result of the chlamydial suspension quantified with 617 IFU/ml was light pink, and might be considered positive (OD_512_ = 0.162). Meanwhile, the result of the chlamydial suspension was colourless if quantified with 126 IFU/ml, and might be considered negative (OD_512_ = 0.098). Therefore, the LOD was 617 IFU/ml for *C. trachomatis*. In addition, the LOD was 10^-3^ u/ml (OD_512_ = 0.155) for α-D-mannosidase.

## Discussion

There are various well-known methods for detecting *C. trachomatis*, including cell culture-, immunology-, molecular biology-, and biochemistry-based methods. Cell culture is complicated to perform and requires experience to produce accurate results, and it also has more stringent requirements for the sampling swabs and transporting before inoculation [21,22]. Therefore, cell culture is rarely used in clinics. Among the available immunological methods, serological tests for the *C. trachomatis* antibody have significant limitations [23], but methods for *C. trachomatis* antigen detection (mainly referring to the lipopolysaccharide, LPS), especially immunochromatography-based methods, have been widely used due to their simplicity of operation. There are two ways to extract the LPS antigen for *C. trachomatis* immunochromatographic assays: heat extraction and acid extraction. Neither of these methods guarantees the full extraction of LPS as an intact antigen, which influences the sensitivity of this method. The biochemical detection of glycogen in *C. trachomatis* inclusions [24] is greatly affected by *Candida spp*. that often exist in these specimens and are especially common during the female menstrual cycle and pregnancy. With the increasing glycogen in vaginal epithelial cells, this method may also cause false positives. The detection of *C. trachomatis* in the United States and Europe has mainly focused on molecular biology methods [25-30]. Although these methods are both high sensitivity and high specificity, it can be challenging for molecular biology methods to meet the requirements of the actual application in clinical screening.

Enzymatic studies of *C. trachomatis,* especially for enzymes with diagnostic significance, have not been reported in the literature. Previous studies [31] and our research have shown that *C. trachomatis* secretes extracellular enzymes with high α-D-mannosidase activity. Although some organisms used in the study such as *C. albicans* have genes encoding α-1,2-mannosidase [32], α-D-mannosidase activity was invisible by naked eyes with 6-chloro-3-indolyl-α-D-mannoside as substrate. This may be because the extracellular α-D-mannosidase from these organisms is much less or the enzyme activity is relatively low. Previous records [31,33-35] on substrates of α-D-mannosidase mainly involved p-nitrophenol-α-D-mannoside and 4-methylumbelliferyl-α-D-mannoside. However, 6-chloro-3-indolyl-α-D-mannoside is a novel chromogenic substrate, and colour reaction of its chromogen is much more sensitive especially in the case of the presence of an azo reagent such as fast violet B salt.

Our results showed that clinical specimens such as urethral discharge, cervical secretions, and vaginal secretions did not interfere with the chromogenic detection of α-D-mannosidase activity to screen for *C. trachomatis*, although prostate massage liquid produced more false positive results. Some human sperm surface proteins possess α-D-mannosidase activity [36], which may be the reason that prostate specimens produce less reliable results. Serotype D was only one of the most prevalent (11.1%), and no serovar L2 was found in China [37]. Although the results of this study suggested that other serotypes, such as serotypes E, F, G, K, H, J, I, and Ba, at least most of them, might have α-D-mannosidase activity, there seems to be some limitation of tests on *C. trachomatis* cultures based on only one strain of serotype D. Further studies and more comprehensive clinical evaluations should be conducted due to little research on α-D-mannosidase activity of *C. trachomatis*. In addition, our studies did not evaluate *C. pneumoniae* or *C. psittaci*; the α-D-mannosidase activities of these species should be studied as well.

## Conclusions

The present study demonstrated that there were no significant differences between the enzymatic method and the reference method when prostate specimens were excluded. Therefore, α-D-mannosidase activity may be a useful marker for *C. trachomatis* in urogenital tract specimens, with many advantages, such as its speed, ease of use, convenience, and need for no special equipment. Taken together, these data show that the enzymatic method has great potential as a clinical method for *C. trachomatis* screening.

## Competing interests

The authors declare that they have no competing interests. We are applying for one patent (CN patent 102286608A) relating to the content of the manuscript, and the authors do not have any objection on the patent right of authorship and ownership.

## Authors’ contributions

ZYW conceived of the study and designed all the experiments and drafted the manuscript. ZYW, GYF, SMW and DCQ performed the experiments. ZYW and ZQW performed the statistical analysis. ZQW and JC provided valuable insight for designing the study and revising the manuscript. All authors read and approved the final manuscript.

## Pre-publication history

The pre-publication history for this paper can be accessed here:

http://www.biomedcentral.com/1471-2334/13/36/prepub

## References

[B1] Centers for Disease Control and Prevention Sexually transmitted disease surveillance, 2009 2011 Atlanta, GA: Centers for Disease Control and Prevention

[B2] Madeleine MM Anttila T Schwartz SM Saikku P Leinonen M Carter JJ Wurscher M Johnson LG Galloway DA Daling JR Risk of cervical cancer associated with Chlamydia trachomatis antibodies by histology, HPV type and HPV cofactors Int J Cancer 2007 120 650 655 10.1002/ijc.22325 17096345 PMC4049152

[B3] Smith JS Bosetti C Muñoz N Herrero R Bosch FX Eluf-Neto J Meijer CJ Van den Brule AJ Franceschi S Peeling RW Chlamydia trachomatis and invasive cervical cancer: A pooled analysis of the IARC multicentric case–control study Int J Cancer 2004 111 431 439 10.1002/ijc.20257 15221973

[B4] Joki-Korpela P Sahrakorpi N Halttunen M Surcel HM Paavonen J Tiitinen A The role of Chlamydia trachomatis infection in male infertility Fertil Steril 2009 91 1448 1450 10.1016/j.fertnstert.2008.06.051 18706556

[B5] Malik A Jain S Rizvi M Shukla I Hakim S Chlamydia trachomatis infection in women with secondary infertility Fertil Steril 2009 91 91 95 10.1016/j.fertnstert.2007.05.070 18635168

[B6] Blas MM Canchihuaman FA Alva IE Hawes SE Pregnancy outcomes in women infected with Chlamydia trachomatis: a population-based cohort study in Washington State Sex Transm Infect 2007 83 314 318 10.1136/sti.2006.022665 17344249 PMC2598687

[B7] Medina M Moya M Hidalgo L Calle A Terán Eui P Molecular identification of endocervical Chlamydia trachomatis infectionamong gestations at risk for preterm birth in Ecuador Arch Gynecol Obstet 2009 279 9 10 10.1007/s00404-008-0647-y 18414882

[B8] Silveira MF Ghanem KG Erbelding EJ Burke AE Johnson HL Singh RH Zenilman JM Chlamydia trachomatis in fection during pregnancy and the risk of preterm birth:a case–control study Int J STD AIDS 2009 20 465 469 10.1258/ijsa.2008.008388 19541887

[B9] Wilkowska-Trojniel M Zdrodowska-Stefanow B Ostaszewska-Puchalska I Redźko S Przepieść J Zdrodowski M The influence of Chlamydia trachomatis infection on spontaneous abortions Adv Med Sci 2009 54 86 90 19403438 10.2478/v10039-009-0008-5

[B10] Rours IG Hammerschlag MR Ott A De Faber TJ Verbrugh HA de Groot R Verkooyen RP Chlamydia trachomatis as a cause of neonatal conjunctivitis in Dutch infants Pediatrics 2008 21 e321 e326 18245405 10.1542/peds.2007-0153

[B11] Black CM Current methods of laboratory diagnosis of Chlamydia trachomatis infections Clin Microbiol Rev 1997 10 160 184 8993862 10.1128/cmr.10.1.160 PMC172947

[B12] Manafi M Fluorogenic and chromogenic enzyme substrates in culture media and identification tests Int J Food Microbiol 1996 31 45 58 10.1016/0168-1605(96)00963-4 8880296

[B13] Orenga S James AL Manafi M Perry JD Pincus DH Enzymatic substrates in microbiology J Microbiol Methods 2009 79 139 155 10.1016/j.mimet.2009.08.001 19679151

[B14] Van Winkelhoff AJ Clement M De Graaff J Rapid characterization of oral and nonoral pigmented Bacteroides species with the ATB anaerobes ID system J Clin Microbiol 1988 26 1063 1065 3290235 10.1128/jcm.26.5.1063-1065.1988 PMC266523

[B15] Wohlsen TD Comparative evaluation of chromogenic agar CM1046 and mFC agar for detection of E. coli and thermotolerant coliform bacteria from water samples Lett Appl Microbiol 2011 53 155 160 10.1111/j.1472-765X.2011.03086.x 21585405

[B16] Wang ZY Kou J Cui J Wang ZQ Chlamydia diagnosis method and reagent 2011 China: CN patent 102286608A

[B17] Taylor-Robinson D Thomas M Dawson PL The isolation of T-mycoplasmas from the urogenital tract of bulls J Med Microbiol 1969 2 527 533 10.1099/00222615-2-4-527 5404806

[B18] Berg ES Anestad G Moi H Størvold G Skaug K False- negative results of a ligase chain reaction assay to detect Chlamydia trachomatis due to inhibitors in urine Eur J Clin Microbiol Infect Dis 1997 16 727 731 10.1007/BF01709252 9405941

[B19] Jang D Sellors JW Mahony JB Pickard L Chernesky MA Effects of broadening the gold standard on the performance of a chemiluminometric immunoassay to detect Chlamydia trachomatis antigens in centrifuged first void urine and urethral swab samples from men Sex Transmitted Dis 1992 19 315 319 10.1097/00007435-199211000-00003 1492256

[B20] Lee HH Chernesky MA Schachter J Burczak JD Andrews WW Muldoon S Leckie G Stamm WE Diagnosis of Chlamydia trachomatis genitourinary infection in women by ligase chain reaction assay of urine Lancet 1995 345 213 216 10.1016/S0140-6736(95)90221-X 7823713

[B21] Mahony JB Chernesky MA Effect of swab type and storage temperature on the isolation of Chlamydia trachomatis from clinical specimens J Clin Microbiol 1985 22 865 867 3902883 10.1128/jcm.22.5.865-867.1985 PMC268547

[B22] Mårdh P Zeeberg B Toxic effect of sampling swabs and transportation test tubes on the formation of intracytoplasmic inclusions of Chlamydia trachomatis in McCoy cell cultures Br J Vener Dis 1981 57 268 272 6268242 10.1136/sti.57.4.268 PMC1045939

[B23] Ngeow YF Limitations of serodiagnosis in chlamydial genital tract infections Ann Acad Med Singapore 1996 25 300 304 8799029

[B24] Wang ZY Fu GY Chen RF Qin Y Fast diagnosis reagent for genital tract Chlamydia trachomatis 2005 China: CN patent 1217004C

[B25] Cheng A Qian Q Kirby JE Evaluation of the Abbott RealTime CT/NG assay in comparison to the Roche cobas amplicor CT/NG assay J Clin Microbiol 2011 49 1294 1300 10.1128/JCM.02595-10 21325546 PMC3122843

[B26] Cosentino LA Landers DV Hillier SL Detection of Chlamydia trachomatis and Neisseria gonorrhoeae by strand displacement amplification and relevance of the amplification control for use with vaginal swab specimens J Clin Microbiol 2003 41 3592 3596 10.1128/JCM.41.8.3592-3596.2003 12904360 PMC179790

[B27] Jalal H Al-Suwaine A Stephen H Carne C Sonnex C Comparative performance of the Roche COBAS Amplicor assay and in-house real-time PCR assay for diagnosis of Chlamydia trachomatis infection J Med Microbiol 2007 56 320 322 10.1099/jmm.0.46762-0 17314360

[B28] Kerndt P Ferrero DV Aynalem G Monga D Wang S Zhang N Wong C Liggins M Meng Q First report of performance of the versant CT/GC DNA 1.0 Assay (kPCR) for detection of Chlamydia trachomatis and Neisseria gonorrhoeae J Clin Microbiol 2011 49 1347 1353 10.1128/JCM.01634-10 21307209 PMC3122860

[B29] Moller JK Pedersen LN Persson K Comparison of the Abbott RealTime CT new formulation assay with two other commercial assays for detection of wild-type and new variant strains of Chlamydia trachomatis J Clin Microbiol 2010 48 440 443 10.1128/JCM.01446-09 20007394 PMC2815632

[B30] Pedersen LN Pødenphant L Møller JK Highly discriminative genotyping of Chlamydia trachomatis using omp1 and a set of variable number tandem repeats Clin Microbiol Infect 2008 14 644 652 10.1111/j.1469-0691.2008.02011.x 18558936

[B31] Greenwell P Kakourou G Raghooputh S Analysis of glycosidases activity in Chlamydia trachomatis L2 Serotype Int J med Update 2006 1 25 32

[B32] Siriwardena A Strachan H El-Daher S Way G Bryan W Glushka J Moremen K Boons GJ Potent and selective inhibition of class II α-D-mannosidase activity by a bicyclic sulfonium salt ChemBioChem 2005 6 845 848 10.1002/cbic.200400397 15800866

[B33] Skudlarek MD Orgebin-Crist MC Effect of swainsonine on rat epididymal glycosidases J Reprod Fert 1988 84 611 617 10.1530/jrf.0.0840611 3143832

[B34] Tulsiani DR Glycan-modifying enzymes in luminal fluid of the mammalian epididymis: an overview of their potential role in sperm maturation Mol Cell Endocrinol 2006 250 58 65 10.1016/j.mce.2005.12.025 16413674

[B35] Vázquez-Reyna AB Balcázar-Orozco R Flores-Carreón A Biosynthesis of glycoproteins in Candida albicans: biochemical characterization of a soluble alpha-mannosidase FEMS Microbiol Lett 1993 106 321 325 8454197 10.1111/j.1574-6968.1993.tb05983.x

[B36] Tulsiani DR Skudlarek MD Orgebin-Crist MC Human sperm plasma membranes possess alpha-D-mannosidase activity but no galactosyltransferase activity Biol Reprod 1990 42 843 858 10.1095/biolreprod42.5.843 2116923

[B37] Gao X Chen XS Yin YP Zhong MY Shi MQ Wei WH Chen Q Peeling RW Mabey D Distribution study of Chlamydia trachomatis serovars among high-risk women in china performed using PCR-restriction fragment length polymorphism genotyping J Clin Microbiol 2007 45 1085 1089 10.1128/JCM.02076-06 PMC1865819 17301282

